# Attracting Israeli nursing students to community nursing

**DOI:** 10.1186/s13584-020-00400-6

**Published:** 2020-10-16

**Authors:** Yael Sela-Vilensky, Keren Grinberg, Rachel Nissanholtz-Gannot

**Affiliations:** 1 Nursing Department, Faculty of Social and Community Sciences, Ruppin Academic Center, Emeq-Hefer, Israel; 2 Department of Health System Management, Ariel University, Ariel, Israel; 3 Myers-JDC-Brookdale Institute, Jerusalem, Israel

**Keywords:** Curriculum, Community nursing, Students, Nursing programs, Clinical placement

## Abstract

**Background:**

The shift from inpatient care to community patient care has had an essential impact on the nursing profession. Despite the growing demand for community nurses in many countries, their number remains relatively low and many students do not perceive this field as an interesting career to pursue. In this review we aimed to understand if exposure of undergraduate nursing students to various nursing work settings during their studies affects their work setting choices after graduation.

**Methods:**

A literature search of papers relating to work setting preferences of nursing students in Israel and other countries was performed. Israel Ministry of Health, Nursing Administration documents and other related documents were also reviewed, with a focus on the nursing training program in Israel.

**Findings:**

While most first-year nursing students have limited knowledge regarding the profession, in later years, their preferences for post-graduation work settings are affected by their exposure to the various clinical fields through knowledge gained in courses together with clinical practice placements. In Israel, specific classroom courses in community nursing are allocated only 6% of the total time allocated to all classroom courses in nursing, and a single clinical placement in community nursing takes place during the third or fourth year of the nursing program, exposing students to a single aspect of community nursing during their nursing training. Studies in other countries have reported that students’ experience during clinical placement contributes to shaping students’ opinions of nurses’ roles within that field. Nursing students who had a primary healthcare placement showed greater intention for working in this setting after graduation.

**Conclusions:**

The lack of exposure to the various aspects of community nursing during undergraduate studies contributes to a lack of motivation for entering this field. Therefore, a profound change is needed in nursing training programs’ curricula to prepare graduates to face future challenges in community nursing. Whilst both hospital and community nursing are equally important, nursing leaders and policy makers must be made aware of the various factors that contribute to new registered nurses’ preferences of hospital over community nursing and build strategies for directing nurses to work in the community in order to respond to the expected nurse shortage in this setting.

## Introduction

Since the 1990s many models have been developed to create conceptual and practical frameworks for integrated chronic patient care in the community, including the Chronic Disease Prevention and Management model [1] and the Chronic Care model [2]. Many healthcare organizations have implemented chronic disease management programs to improve the quality of patient care in the community [3–6]. According to the Israeli Ministry of Health’s approach, the community is the natural place for providing most of the health services to Israel’s citizens [7].

Community nursing (also called public healthcare nursing) allows nurses to treat patients within their natural living environment. Such services are also less expensive than inpatient care, thereby helping to reduce public spending and the burden on the health system [8]. Nurses that provide community health services play key roles in prevention of disease and injury, alleviation of disability, health promotion and medical education. They also manage and provide care and follow-up across a broad range of settings [9–11]. Community nurses devote a great deal of their time to instructing individual patients (e.g. how to inject insulin) and patient groups (e.g. weight reduction groups). Moreover, nurses’ authorities and roles in the healthcare system have greatly expanded due to the delegation of roles previously held by doctors, such as counseling, training, coordination and management of care [12, 13]. These roles position nurses as initiators and carers. At the two largest health funds in Israel, community nurses are case managers of patients with chronic diseases: they maintain continuous contact with patients, encourage them to perform relevant tests and comply with treatments, and refer them to a physician when necessary. The nurses also create an annual intervention plan for each patient and implement it [14].

However, despite the growing demand for community nurses in many Western countries, there is a significant shortage of nurses who work in this setting due to the pending retirement of older nurses, job discontent, and challenges in attracting new nurses and retaining nurses in this field. In addition, many nursing students do not perceive this field of nursing as an interesting career to pursue [15–19]. In the United States the reason for such shortage in community or public health nurses include lower salaries that were traditionally paid to nurses by governmental agencies compared to major health care facilities such as hospitals. Furthermore, community healthcare was perceived as having a “low status” with a smaller variety of treatments and opportunities for professional development [20]. In this review we aimed to understand if exposure of undergraduate nursing students to nursing work settings during their studies affects their work setting choices after graduation.

## Methods

We performed a literature search of papers relating to work setting preferences of nursing students in Israel and other countries. In addition, Israel Ministry of Health, Nursing Administration documents and other related documents were reviewed, with a focus on the nursing training program in Israel.

## Findings

### Nurse staffing standards in Israel

In Israel, approximately 70% of nurses work in hospitals, while only 20% work in community care settings. The rest are employed in research, nursing training institutions, universities or hold administrative positions [21, 22]. Nirel et al. analyzed several demand and supply projection models and found that there is a considerable gap between the supply and demand for registered nurses, which is expected to increase over time up to 2030 [23]; However, unlike other Western countries, in Israel there is no re-registration mechanism for licensed nurses; therefore it is not possible to obtain regular updated information on nurses’ occupational characteristics [22].

The size of the nursing workforce in Israeli hospitals (inpatient and outpatient wards) is determined by a fixed ratio of nursing positions (nurses and auxiliary staff) per bed, which was set by a collective agreement between the public employers and the Nurses’ Union in 1997. The agreement stipulates the ratio of nursing positions per bed without distinction between nurses and auxiliary staff; however, the mechanism for updating these standards was not adapted for medical and technological advancements over the years, resulting in nurse staffing deficiencies [12]. The Ministry of Health has acknowledged these deficits and the growing gap between the system’s needs and the actual staffing standards [21].

In contrast to nursing staffing standards in hospitals, only one of the 4 Israeli health funds has nursing staffing standards for health services that are provided in the community. Rather, the number of nursing positions is determined by each health fund according to its work plan, services, and service levels that it would like to provide to its members within its budget. The nursing staffing standard for health services provided directly by the Ministry of Health or by secondary providers (e.g. family health clinics run by local municipalities) is determined by a fixed ratio of the number of individuals requiring the service per nursing position [24].

The lack of uniformity in nursing staffing standards among health funds makes it difficult to quantify the shortage of nurses in the community. However, it is clear that the overall shortage of nurses together with population growth, particularly the aging population, the transfer of care of complex patients to the community, and the expansion of nurses’ roles, all mean that there is a need to increase the number of nurses working in the community [24]. Nirel et al. estimated that by 2030, approximately 8950 nurses would be needed in health funds and public health services compared to 6770 in 2010, representing a 32% increase in the number of nurses required in the community [24]. In a study that evaluated the changes and processes in community nursing through interviews of nurses and nursing managers, the dearth of resources and, in particular, the lack of human resources and nursing staffing standards was perceived by nurses as the main barrier to the development of community nursing in Israel [25].

### Nursing students’ preferences for work settings

Upon starting their studies most nursing students’ knowledge about the profession is limited and is often affected by the way it is portrayed in the media – on television and the internet [26–28]. In a study that examined nursing leaders’ perceptions and attitudes regarding the career preferences of nursing students, most participants agreed that the media has a major impact on shaping students’ perceptions in that most media exposure related to nursing, and specifically when advertising nursing training programs, is given to work within hospital departments, such as intensive care and trauma units. The use of technology within these departments is presented as exciting and challenging, compared to the role of the nurse in the community which is hardly ever presented [29].

As the students advance in their years of study, they gain ideas about the various possibilities of the profession and form opinions about how they see their future career and nursing specialties. Many studies have shown that nursing students’ choices of their post-graduation work setting are affected by their exposure to the various clinical fields through knowledge gained in courses together with clinical practice placements [16, 30–34].

Most nursing students do not regard working in community healthcare appealing [15, 17, 31, 35], probably due to a limited understanding of the nurse’s role in this setting [20, 36]. Instead, they prefer working in hospitals because they perceive this work setting as more challenging because they believe it involves more complex techniques compared to community nursing [34, 35, 37, 38]. This inclination for preferring hospital nursing over community nursing was already seen among first-year nursing students [38–40]. In a study conducted in England, nursing students preferred placements that offered an opportunity to practice clinical skills and showed distinct preferences for certain types of placement areas, such as cardiology and intensive care, but were not so enthusiastic about placements such as care of the older adult [41].

Although students’ career preferences during their pre-graduate studies were not static with regards to their preference for certain clinical fields [34], the preferences of most students for working in hospital settings did not change by the end of their studies [34, 40]. In a study conducted among nursing students in Israel, the decision to work in a hospital was noted as more important than choosing a specific clinical field. The students indicated that in the first years after graduation, they prefer working in hospitals over community setting in order to gain professional experience. Community nursing was also perceived as more monotonous than nursing in hospitals [42]. The belief that hospital experience is essential in the development of confidence in their nursing skills and clinical judgments before working in general practice was also observed among Australian nursing students [36].

The desire of nursing students to work in primary healthcare was found to be associated with older age, greater perceived value of employment conditions including flexibility, and less perceived importance of workplace support [43].

An additional factor that affects students’ choice of the hospital setting over the community is the population of patients treated in the community. Several studies from various countries have shown that registered nurses and nursing students prefer working with younger people who can recover over elderly individuals [44–46]. Happel [45] reported that working with older people was the least preferred area of practice for students, with declining popularity during the training program. Some nursing students regarded caring for older adults as unsatisfying, depressing, boring and frightening [47]. The elderly were also viewed as an obstacle and burden [48]. However, knowledge of aging and a preference for working with the elderly was associated with positive attitudes towards older people [48].

In Israel, students’ attitudes to nursing were examined in relation to the concept of the geriatric field as a future field of practice. The results showed that only 12% of students would consider working in this field while over 60% of the study participants are not at all interested in this area and 27% would consider working in the field only after appropriate training. The variables predicting intentions to engage in geriatrics included expanding nurses’ authority as a clinical specialist, positive attitudes toward the elderly, and prior experience in geriatric care. Training in an academic nursing program was found to be a negative predictor [21].

### The nursing training curriculum in Israel

In Israel there are several non-academic (2.5–3 years-long) and academic (4 years) nursing training programs. These programs are based on the Nursing Administration’s core curriculum, which consists of 126 credits (2648 h), two-thirds of which are classroom courses, and about one-third of practical (clinical) studies. The curriculum is extensive and includes a basic science division (462 h), a nursing science division (210 h), a clinical studies division comprising classroom courses (728 h), clinical placement (960 h), and internship (288 h).

In addition to teaching the Nursing Administration’s core curriculum, each nursing program administrator may add various additional contents. Completion of the core program constitutes the minimal standard for registering at the Ministry of Health. To obtain an RN registration, the nurse must also successfully pass the governmental exam in nursing.

A nurse who graduates from the core program is defined as an “incipient” nurse and can work in almost all departments, but her authorities are limited to specific activities. Upon completion of the core program, nurses start an internship in their clinical specialization and can start working as a nurse. RNs with an academic degree in nursing can take additional courses in order to expand their responsibilities and authorities. These courses allow the nurses to specialize in specific clinical fields in order to expand their authorities within the field. The nurses must pass an exam at the end of the course to obtain certification [49].

Within the nursing training programs, classroom courses in specific clinical fields are intended to prepare the students for clinical placement in that field, which begins during the second year of the program. These courses focus on the traditional learning of diagnosis and treatment in particular diseases and do not emphasize the holistic aspects of prevention and health promotion, and their broad contexts - which in practice constitute a large part of the nurse’s role. In a study that described the changes in contemporary nurses’ roles in Israel, Nissanholtz-Gannot et al. reported that current nursing programs are more academic and less focused on teaching caring skills, so that a nurse that completes her studies today has less caring skills than those of nurses who completed their studies in the past [25].

Practical clinical placement comprises approximately one third of the total number of hours dedicated to nurse training and it allows the students to apply the theories they acquired in the classroom [50]. Specifically, clinical placement [51, 52]:

- Enables students to practice clinical skills and nursing techniques and procedures in a variety of treatment settings and function in authentic situations.
- Exposes students to the reality of working as a nurse.
- Provides conflicts with the theories learned in the classroom and their application in the field within time and resource constraints.

Table 1 shows the number of hours allocated to classroom courses and clinical placements in each of the nursing specialties in Israel [53]. Notably, classroom courses in community nursing are allocated only 42 h (3 credits), which are 6% of the total time allocated to classroom courses in nursing. A single clinical placement in community nursing takes place during the third or fourth year of the nursing program and constitutes 15% of the 960 h comprising clinical placements. In this single clinical placement in community nursing, the student is exposed to work either within a primary clinic or at “Tipat Halav” (health centers that provides public health services to pregnant women, babies, children, and their families), but not both. As such, the students are only exposed to a single aspect of community nursing during their nursing training.

**Table 1:** Clinical studies unit: the number of hours allocated to each nursing specialty in the nursing curriculum [53]

	Classroom courses		Clinical placement		Total	
Subject	Hours	Credits	Hours	Credits	Hours	Credits
**Internal Medicine**	168	12	192	4	360	16
**Surgery**	168	12	192	4	360	16
**Obstetrics and gynecology**	98	7	144	3	242	10
**Pediatrics and adolescents**	98	7	96	2	194	9
**Geriatrics**	28	2	–	–	28	2
**Community**	**42**	**3**	**144**	**3**	**186**	**6**
**Emergency and traumatology**	28	2	96	2	124	4
**First aid and emergency situation**	28	2			28	2
**Total**	**728**	**52**	**960**	**20**	**1688**	**72**

### Directing the nursing curriculum for work in community settings

The increasing demands for community nurses points to a gap between the classroom courses taught in nursing programs, which remain focused on care within the hospital environment and the work expected of nursing graduates in community healthcare [54, 55].

To reflect the changes in health care provision, the Association of Community Health Nursing Educators (ACHNE) has recommended that nursing curricula include broad clinical training that will reflect the nurse’s growing involvement in the community at all levels [56–58]. The Institute of Medicine has recommended that nurses should be provided with tools to plan, implement, and evaluate strategies for prevention, protection, and preservation of patient and community health. Such tools would enable nurses to promote and maintain patients’ health in their natural environment, preventing illnesses, disabilities and injuries [59]. The curriculum should also emphasize prevention and health promotion issues, chronic care in the community and end of life [60]. In a study conducted among nursing leaders and policymakers in Israel, some of the interviewees stated that the generic training in community nursing in Israel does not enable nursing students to see the variety of opportunities available for nurses who work in community healthcare. As a result, students develop a limited worldview of nurses’ role in this setting [29].

Students’ experience during clinical placement – whether positive or negative - contributes to shaping the student’s opinion of the nurse’s role within that field [61]. If students perceive their experience as negative they are unlikely to want to work in this field in the future, while positive experiences can lead the student to choose this area as a future field of practice [16, 32, 62]. Bloomfield et al. [43] reported that nursing students who had a primary healthcare placement showed greater intention for working in this setting after graduation. In addition, the length of clinical placement has been identified as important to nursing students [63]; therefore, placement must be long and significant enough to change the student’s perception of the nurse’s role in the community.

It is important to note that several studies related to the issue of the need for support during community clinical placement – both from the students’ perspective as well as from their supervisors’ (also referred to as mentors or preceptors) perspective. Anderson and Kiger [64] reported that students greatly appreciated the opportunity to work independently in the community during clinical placements. The students reported increased confidence. They felt that they developed skills in communication and therapeutic patient relationships, obtained experience in managing patients and were a valued member of a team [64]. According to McInnes et al., students regarded their community placement less positively if they were given fewer occasions to practice their clinical skills, or if they felt that they were not accepted into the cultural entity of the primary care facility [36]. Murphy et al. reported that students had marked preferences for particular types of placements appearing to favor those that may offer more one- on- one relationship with trained nurses with the opportunity to master clinical skills to facilitate their acculturation into nursing [41]. From the supervisors’ point of view, in Sweden, supervisors in community placements felt that universities did not support them sufficiently and demanded better support and cooperation from nursing education stakeholders [65]. In Australia, nurse mentors were satisfied with mentoring nursing students in their setting, but lack of practice payments and time were noted as key barriers to student placements [66].

In addition to focusing on the care of individuals and their families, clinical placement in the community should be expanded to working with specific patient populations including, the elderly, mental health patients and high-risk communities. Nursing curricula in Australia, Canada and the United States have extended nursing students’ clinical placements to experience in remote areas [67–69].

### Implications for policy changes

To encourage nursing students to choose community nursing, we suggest increasing students’ exposure to community work by integrating community-focused content into nursing training programs. Within the core curriculum, more hours should be allocated for studying community nursing and showing that these are not separate content worlds but an integral part of the overall approach to patient care and expectations of nurses. Bouchaud and Gurenlian suggest that in order to integrate the “community” within the nursing curriculum without separating it from hospital nursing, aspects of community nursing should be incorporated into each course of the nursing curriculum [55]. Clinical placement in the community must be expanded to expose students to the diversity of nursing roles in the community, and to emphasize the challenges of treating elderly patients, providing home care and mental health aspects involved in community nursing.

Holistic aspects of prevention and health promotion should be emphasized, rather than focusing only on diagnosis and treatment. The National Committee of the Council of Higher Education recommended already in 2010 to reduce teaching by physicians, to teach less about medical models and to expand elective courses that focus on human aspects, as well as to devote more courses to group work. Although there is some recognition of the need to move from a traditional medical model to a focused core competencies model, efforts in this direction are mostly localized.

Finally, nursing leaders should ensure that community nursing is perceived as a positive and interesting career option. Therefore, the identity of community nursing should be strengthened, and the visibility of community nursing should be enhanced from among other nursing roles, specifically those in desired hospital departments. In addition, efforts to recruit nurses to the community at various stages of their career should be made in order to reach nurses who started their career in hospitals.

## Conclusion

The lack of exposure to the various aspects of community nursing during undergraduate studies results in a lack of motivation for entering this field. Therefore, a profound change is needed in nursing training programs’ curricula in order to prepare graduates to face future challenges in community nursing. Whilst both hospital and community nursing are equally important, nursing leaders and policy makers must be made aware of the various factors that contribute to new registered nurses’ preferences of hospital over community nursing and to build strategies for attracting nurses to work in the community in order to respond to the expected nurse shortage in this setting.

## Data Availability

Not applicable.
